# Cultivar-dependent interplay between volatile biosynthesis and texture deterioration in pear during cold storage and shelf-life

**DOI:** 10.1016/j.fochx.2026.103837

**Published:** 2026-04-05

**Authors:** Guanwei Gao, Chen Yin, Luming Tian, Haifei Li, Hongliang Huo, Dan Qi, Ying Zhang, Chao Liu

**Affiliations:** a Institute of Pomology, Chinese Academy of Agricultural Sciences, Xingcheng 125100, China; b Key Laboratory of Germplasm Resources Utilization of Horticultural Crops, Ministry of Agriculture and Rural Affairs, Xingcheng 125100, China; c Laboratory of Quality & Safety Risk Assessment for Fruit, Xingcheng 125100, China

**Keywords:** Pear, Volatile organic compounds, Soluble solids content, Texture, Storage

## Abstract

Pear aroma quality is predominantly governed by volatile organic compounds (VOCs), which are critically influenced by postharvest storage conditions. This study tracked VOCs, soluble solids content (SSC), and texture dynamics in Nanguoli, Jingbaili, and Korla pears during 105-day cold storage and a 15-day shelf life. A total of 301 VOCs were identified, with esters increasing dramatically (25.96- to 113.55-fold) after 75-day cold storage, especially in soft-fleshed cultivars. Texture declined in these cultivars during shelf life, whereas Korla pears maintained exceptional firmness (> 90% retention) and stable texture parameters. Multivariate analysis revealed a strong negative correlation (*r* < −0.8) between ester accumulation and textural integrity, highlighting a fundamental trade-off between tissue softening and aroma development. Furthermore, up to 85 VOCs were identified with variable importance in projection scores > 1. These compounds may help clarify the mechanistic basis of texture deterioration and inform strategies for postharvest quality preservation in future studies.

## Highlights

• Esters content in pears increased 26–114 fold during 75-day cold storage.
• Soft-fleshed pears had more esters, crisp-fleshed pears retained firmness.
• Ester accumulation strongly correlated with texture degradation (*r* < −0.8).
• 85 key VOCs discriminated pear texture types via OPLS-DA model.
• VOCs profiles and texture changes were cultivar-dependent during storage.

## 1 Introduction

Pear (*Pyrus*) is one of the most economically significant temperate fruits around the globe, valued for its nutritional and sensory qualities (Wu et al., 2018). As a critical quality attribute of fruit, aroma directly influences consumer acceptance and market competitiveness (Lytou et al., 2019). It is primarily determined by the composition and concentration of volatile organic compounds (VOCs). In pear fruits, more than 335 VOCs have been identified (Qin et al., 2012; Shi et al., 2025). Pear aroma arises from a complex mixture of VOCs, predominantly esters, aldehydes, alcohols, ketones, and terpenoids. According to previous studies (Guan et al., 2025; Soomro et al., 2023; Wang et al., 2023), composition and concentration of VOCs in fruit are mainly influenced by genetic attributes, growing conditions, postharvest treatments and storage conditions.

Postharvest storage is essential for prolonging fruit availability and preserving quality (Rajapakshe et al., 2025). Low-temperature storage effectively inhibits physiological deterioration and pathogen growth in fruit (Dai et al., 2025). Meanwhile, shelf life under ambient conditions governs ripening progression and aroma development prior to consumption. Therefore, understanding the dynamic changes in the profiles of VOCs in fruit across these phases is vital for optimizing storage strategies. Although previous studies have reported changes in the groups of VOCs in pear fruits during storage (Zhang & Yin, 2023), comprehensive analyses across multiple cultivars and storage durations remain limited.

In addition to aroma, soluble solids content (SSC) and textural properties are fundamental quality parameters that determine fruit acceptability and market value (Cao et al., 2024; Vu et al., 2023). SSC is a key indicator of sweetness, reflecting sugar accumulation, and it influences the taste and nutritional value for fruits (Wang et al., 2025). Texture, including firmness and chewiness, affects mouthfeel, storability, and processing suitability of fruit (Ross et al., 2023). Metabolic connections between VOCs and these quality traits are biologically plausible, given their shared precursors and the influence of cellular structure on volatile release. Their interactions during postharvest storage are not well characterized in pears.

This study examined the dynamics of VOCs, SSC, and textural properties in three economically important Chinese pear cultivars, Nanguoli (*Pyrus ussuriensis*), Jingbaili (*Pyrus ussuriensis*), and Korla pear (*Pyrus sinkiangensis*), during cold storage and a subsequent shelf life at ambient temperature. Nanguoli and Jingbaili pear often require post-ripening before they are suitable for consumption, gradually releasing their aroma. It aimed to clarify the relationships between aroma development and key physicochemical quality attributes, thereby supporting the development of tailored storage protocols and quality-oriented breeding strategies.

## 2 Materials and methods

### 2.1 Reagents and chemicals

High-performance liquid chromatography-grade methyl alcohol and purified water were purchased from Fisher Scientific (Pittsburgh, PA, USA) and Wahaha Foods Co., Ltd. (Hangzhou, China), respectively. Cyclohexanone (> 99%) used as internal standard was prepared in 10% methyl alcohol (*v/v*) and obtained from Tianjin chemical reagent Ltd. (Tianjin, China). Agela Technologies (Newark, DE, USA) provided sodium chloride (NaCl).

### 2.2 Sample storage and collection

Fruit samples of two soft-fleshed Jingbaili, Nanguoli, and crisp-fleshed Korla pear cultivars were obtained from the National Germplasm Repository of Pear and Apple (Xingcheng, China). Uniformly sized fruits at commercial maturity, free from pests and mechanical damage, were selected. These fruits were immediately transferred to cold storage maintained at 0 ± 1 °C for storage periods of 45, 75, and 105 days. After each designated storage interval, fruits were removed and subsequently held at room temperature for 0, 5, 10, and 15 days. All analyses were performed in triplicate.

### 2.3 SSC detection

Ten fruits per variety were selected. After peeling and core-removing, the flesh was sliced, homogenized, and juiced. The extracted liquid was directly applied to a PAL-1 digital refractometer (ATAGO, Tokyo, Japan) for SSC measurement. Ten replicates were performed and the mean SSC was calculated.

### 2.4 Texture polyhedral analysis

The textural properties were evaluated using the Texture Profile Analysis (TPA) method with a TMS-TOUCH food texture analyzer (Food Technology Corporation, USA). The experimental method was adapted from that described by Qiu et al. (2021). Measurements were performed using a P/35 probe (75 mm diameter). The pear fruits were longitudinally bisected along the fruit stalk. From each half, cylindrical samples (10 mm diameter, 9 mm height) were excised from the mesocarp region using a cork borer. Ten replicate samples were randomly selected for each measurement. Test parameters were set as follows: probe return height to sample surface = 10 mm, deformation percentage = 50%, test speed = 30 mm/min, and trigger force = 1 N. The following texture parameters were derived from the resulting force-time curves: fracture force, firmness, cohesiveness, springiness, gumminess, and chewiness. For firmness, TPA was conducted using a dual compression cycle to simulate the mechanical process of mastication. Firmness 1 and Firmness 2 were defined as the peak forces recorded during the first and second compression cycles, respectively.

### 2.5 Sample preparation for VOCs analysis

VOCs in the pear fruits were quantified based on the method described by Gao, Liu, et al. (2022). After the removal of peel and core, the flesh was sectioned using a stainless-steel knife. To prevent enzymatic browning, the sliced flesh was combined with NaCl (1:1, *m/m*) and subsequently homogenized using a commercial juice extractor. Subsequently, a 10.0 g aliquot of the homogenate was transferred into a 20 mL solid-phase microextraction (SPME) vial along with 0.1 mL of cyclohexanone solution (0.2 mg/mL). Purified water was then added to the vial to achieve a total volume of 10 mL prior to being sealed with a screw cap fitted with a PTFE/silicone septum. All prepared samples were stored at −20 °C prior to analysis.

### 2.6 SPME conditions

VOCs were extracted from samples using an AOC 6000 auto-sampler (Shimadzu, Tokyo, Japan). A divinylbenzene/carboxen/polydimethylsiloxane (DVB/CAR/PDMS) SPME fiber (50/30 μm film thickness; Supelco, Bellefonte, USA) was employed for all extractions. Prior to each analysis, the fiber was conditioned at 250 °C for 10 min. To facilitate the release of VOCs, sample vials were incubated at 80 °C for 15 min under constant agitation (300 rpm). Subsequently, the SPME fiber was inserted into the vial headspace, where adsorption was performed for 15 min with continuous heating and agitation.

### 2.7 Gas Chromatography-mass spectrometry (GC–MS) analysis

Qualitative analysis of VOCs was performed using a Shimadzu QP2010 Plus GC coupled to a QP2010 mass spectrometer (Shimadzu, Tokyo, Japan). VOCs adsorbed on the fiber were thermally desorbed in the injection port at 200 °C for 1 min under splitless conditions. Compound separation was achieved on an HP-INNOWAX capillary column (60 m × 0.25 mm × 0.25 μm film thickness; Agilent Technologies). The GC oven temperature program was initiated at 50 °C with a 1-min hold, followed by an increase to 180 °C at a rate of 2 °C/min (held for 1 min), and a final ramp to 230 °C at 10 °C/min with a 10-min hold. Ultra-high purity helium (99.999%) was employed as the carrier gas at a constant flow rate of 1.0 mL/min. Mass spectrometric detection was operated in electron ionization (EI) mode at 70 eV, with scanning conducted across the mass range of *m/z* 50–500. Identification of target compounds was based on comparison of acquired mass spectra with reference spectra in the NIST 17 s library database, requiring a minimum match quality of 90%.

### 2.8 Data analysis

Content of VOCs in sample was quantified using the peak area normalization method, whereby the peak area of each VOCs was compared to that of the internal standard (cyclohexanone). VOCs concentration, expressed in mg/kg fresh weight (FW), was calculated using the formula: VOCs content = (VOCs peak area / internal standard peak area) × internal standard content. Statistical analyses and bar graph generation depicting volatile component number and concentration were conducted using Microsoft Excel 2016 (Redmond, WA, USA). Correlation heatmaps were plotted using Origin 2021 software (OriginLab Corporation, Northampton, MA, USA). Principal component analysis (PCA), hierarchical cluster analysis (HCA), orthogonal projections to latent structures-discriminant analysis (OPLS-DA), and variable importance in projection (VIP) analysis were performed using SIMCA version 14.1 (Umetrics, Umeå, Sweden). Additionally, one-way analysis of variance (ANOVA) was carried out with SPSS version 26.0 (IBM Corporation, Armonk, NY, USA), with a significance level set at *p* < 0.05.

## 3 Results and discussion

### 3.1 Composition of VOCs in pear fruits

In this study, changes in composition of VOCs in pear fruits with three different cultivars, including Nanguoli, Jingbaili, and Korla pear, during cold storage durations and shelf lives, were analyzed using SPME coupled with GC–MS. As depicted in Fig. S1, a total of 301 volatile compounds were identified, comprising 105 esters, 69 alcohols, 44 ketones, 32 aldehydes, 27 hydrocarbons, 10 acids, and 14 miscellaneous compounds. Consistent with prior findings (Gao, Zhang, et al., 2022; Wang et al., 2023; Xu et al., 2023), esters, alcohols, ketones, and aldehydes constituted the predominant groups of VOCs in pear.

**Fig. 1:**
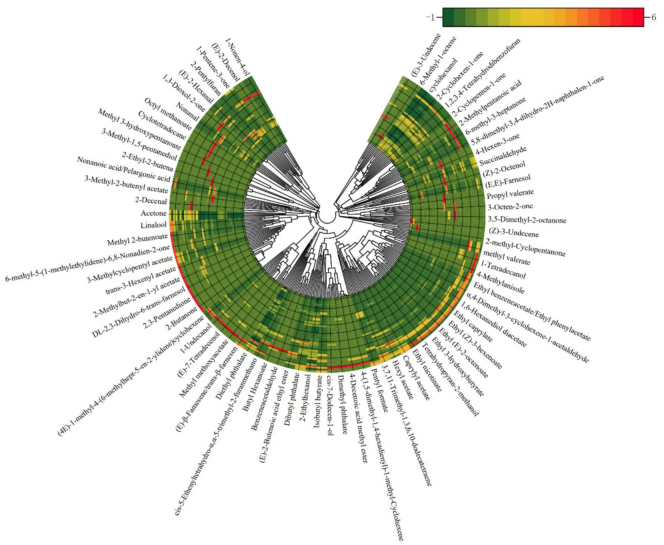
Composition of VOCs in pear fruits with three representative cultivars (Nanguoli, Jingbaili, and Korla pear).

With the aroma of ripe fruits, esters constituted the predominant class of VOCs contributing to pear aroma (Qin et al., 2012). Among them, ethyl butyrate, methyl hexanoate, ethyl hexanoate, butyl acetate, and hexyl acetate consistently exhibited elevated levels across all pear samples (Fig. 1). This finding aligns with previous studies identifying butyl acetate and hexyl acetate as key esters in pear (Dunemann et al., 2012; Shi et al., 2025). In addition, ethyl butyrate, methyl hexanoate, and ethyl hexanoate impart strong fresh apple or pineapple-like fruitiness, while butyl acetate and hexyl acetate deliver the quintessential fresh, sweet aroma of apple, banana and pear.

### 3.2 Comparison of profiles of VOCs in pear fruits with different cultivars

As illustrated in Fig. S2, significant differences existed in types (A) and concentrations (B) of VOCs among Nanguoli, Jingbaili, and Korla pear. Esters, alcohols, aldehydes, and ketones collectively constituted over 75% of the total VOCs in both variety and content, establishing their dominance in volatile profile of pear fruits. As can be seen from Fig. S2A, Jingbaili exhibited the highest diversity of VOCs (227 types), followed by Nanguoli (206 types) and Korla pear (138 types). Consistent with previous studies, esters dominated the aroma composition in both Nanguoli (Zhang & Yin, 2023; Zhou et al., 2015) and Jingbaili pears (Xu et al., 2023), with 82 and 69 types respectively, while alcohols represented the second most abundant group (58 and 51 types, respectively). For Korla pear, alcohols (36 types) constituted the most diverse group of VOCs (Xu et al., 2023), followed by esters (29 types). Ketones, identified as primary contributors to fruity sweet flavors (Qin et al., 2012), constituted the third-largest category of VOCs across all the pear cultivars (20–33 types). Aldehydes, known to impart diverse special aromas even at trace levels (Sun et al., 2010), were predominant in immature fruit. Aldehyde VOCs numbered 16 in Korla pear, 19 in Nanguoli, and 27 in Jingbaili pears.

Consistent with diversity patterns of VOCs, total content of VOCs decreased in the order: Jingbaili (106.1 mg/kg FW) > Nanguoli (99.6 mg/kg FW) > Korla pear (54.2 mg/kg FW) (Fig. S2B). Ketones constituted the predominant class of VOCs in Jingbaili (29.6 mg/kg FW) and Korla pear (14.3 mg/kg FW), followed by alcohols (21.6 and 12.4 mg/kg FW, respectively) and esters (26.7 and 9.8 mg/kg FW, respectively). Notably, esters reached peak concentration in Nanguoli (37.0 mg/kg FW), exceeding those in Jingbaili (26.7 mg/kg FW) and Korla pear (9.8 mg/kg FW). This finding contrasted with our prior study (Gao, Zhang, et al., 2022) identifying aldehydes as the dominant VOCs in Nanguoli. The discrepancy may stem from distinct storage conditions, wherein aldehydes undergo enzymatic conversion to alcohols and subsequent esterification (Shi et al., 2025). Thus, elucidating dynamic changes of VOCs under specific storage regimes is warranted to optimize pear aroma development.

### 3.3 Changes of VOCs in pears during storage

#### 3.3.1 Cold storage

**Fig. 2:**
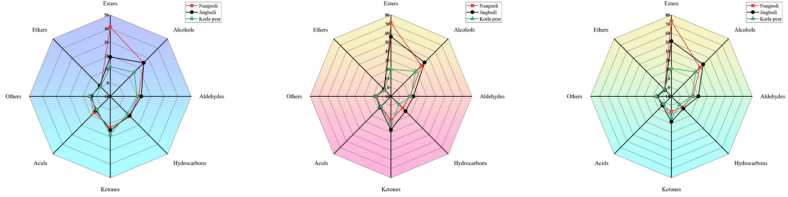
The number of VOCs types in pear fruits after 45, 75 and 105 d of cold storage. Note: cold means cold storage at 0 ± 1 °C; 0–15 d means shelf life at room temperature after cold storage.

Cold storage is an effective and commonly used technique for preserving fruit quality (Liu et al., 2024; Rajapakshe et al., 2025). As shown in Fig. 2, the diversity of VOCs in pear fruits gradually increased during the initial 75 days of cold storage. Notably, number of ester and alcohol compounds increased from approximately 10–45 and 15–25 types to 20–70 and 30–45 types, respectively. Beyond this period, further extension of storage time resulted in minimal change in diversity of VOCs. This acceleration indicated enhanced activity of key enzymes in ester biosynthesis, specifically alcohol acyltransferases (AATs) and reductases acting on lipoxygenase (LOX)-derived aldehydes, under prolonged cold condition (Espino-Diaz et al., 2016; Luo et al., 2021). In addition, Nanguoli pears exhibited the richest variety of ester VOCs. Meanwhile, Jingbaili pears showed the most pronounced increase in both ester and alcohol VOCs during cold storage.

Additionally, after 45 days of cold storage, contents of ester, alcohol, aldehyde, and ketone VOCs in pear fruits ranged between 0 and 0.02, 0.08–0.11, 2.07–3.89, and 0.09–0.10 mg/kg FW, respectively. By day 75, content levels of those compounds increased to 0.33–0.87, 0.13–0.16, 3.80–8.87, and 0.06–0.31 mg/kg FW, respectively. Content of ester VOCs exhibited the most dramatic increase (25.96- to 113.55-fold), far exceeding that of other classes of VOCs. The results demonstrated that ester VOCs are the primary aroma-active compounds in pear fruits and serve as the final conversion products of aromatic substances during storage (Gao, Zhang, et al., 2022). Among them, Nanguoli displayed the greatest magnitude of increase in content of ester VOCs. After 105 days of storage, VOCs content declined markedly in the most cultivars, except for continued increases in esters, alcohols, and aldehydes observed in Nanguoli pears.

#### 3.3.2 Shelf life

**Fig. 3:**
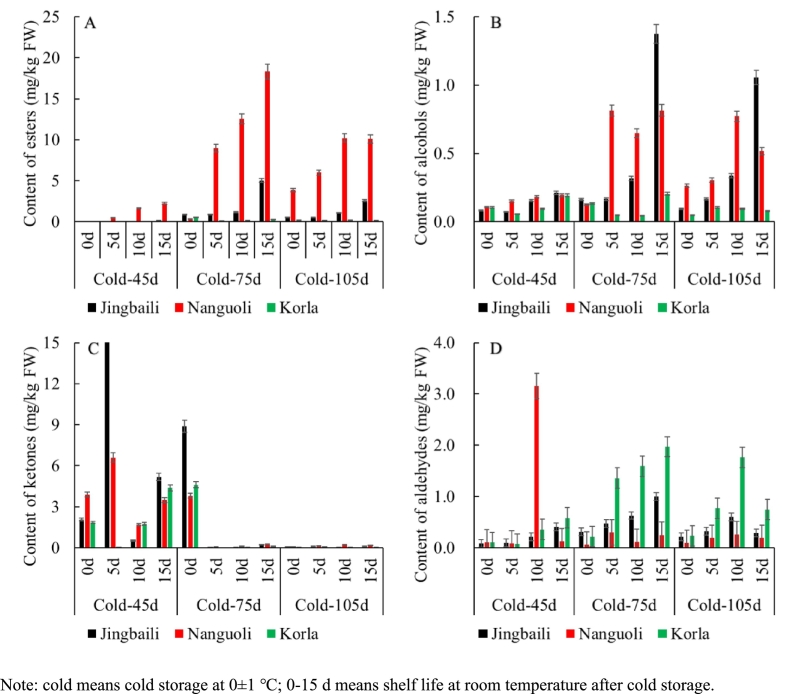
Dynamic changes of VOCs content in Nanguoli, Jingbaili, and Korla pear fruits during storage.

As demonstrated in Fig. 3, as a characteristic climacteric fruit, pear exhibited substantial shifts in its volatile aroma profile during shelf life, especially in the increases of ester, alcohol, ketone, and aldehyde VOCs concentrations. When subjected to 15-day shelf life after 45-, 75-, and 105-day cold storage, ester VOCs in Nanguoli increased from 0, 0.33, and 3.88 mg/kg FW to 2.24, 18.30, and 10.11 mg/kg FW, respectively (Fig. 3A). These elevations in magnitude and rate exceed those recorded in Jingbaili and Korla pears. Notably, ester levels (0.14–12.55 mg/kg FW) measured after 75-day storage combined with shelf life surpassed those under other storage durations (0.03–10.11 mg/kg FW). Results in this work demonstrated that the optimal enzyme induction and substrate accumulation in pear fruits were achieved after 75-day cold storage (Li et al., 2014).

Alcohols in fruit serve as substrates for ester VOCs biosynthesis (Rajendran et al., 2023). In this study, similar dynamics were observed for alcohols and ester VOCs during storage (Fig. 3B). During the initial 0–10 days of shelf life, alcohol content increased from 0.08 to 0.26 to 0.16–0.77 mg/kg FW in both Nanguoli and Jingbaili. By day 15, a further rise to 0.21–1.37 mg/kg FW (the highest among varieties) was recorded in Jingbaili, whereas accumulation in Nanguoli slowed or declined. Korla pear exhibited an initial decline (0.04–0.09 mg/kg FW on day 10), followed by an increase (0.08–0.21 mg/kg FW on day 15). Similar dynamic patterns to ester VOCs were observed that alcohol levels measured after 75-day cold storage (0.21–1.37 mg/kg FW) consistently exceeded those of other treated groups (0.08–1.06 mg/kg FW).

Ketones in pear fruits remained elevated (0.53–16.89 mg/kg FW) after 75-day cold storage or 45-day cold storage plus shelf life but dropped below 0.5 mg/kg FW upon prolonged storage (Fig. 3C). The 45-day cold-stored group showed an initial increase followed by a decrease during shelf life. According to previous studies (Hendges et al., 2018; Zlatić et al., 2016), this dynamic may be regulated by β-ketoacyl-CoA lyase and fatty acid β-oxidation pathways, with extended cold storage potentially inactivating key enzymes.

After harvest, aldehyde VOCs in fruit were typically produced for a period and were even increased under certain conditions. In climacteric fruits at optimal ripeness, aldehyde production (particularly of compounds associated with ripening aromas) was typically enhanced and peaked, contributing to favorable flavor development (Defilippi et al., 2005). In this study, aldehyde VOCs levels in fruits of three pear cultivars were detected at 0.06–0.23 mg/kg FW on day 0 of shelf life and peaked (0.25–3.16 mg/kg FW) on day 10 or 15 (Fig. 3D). The highest aldehyde concentration was observed in Nanguoli (3.16 mg/kg FW), whereas the mean aldehyde content was significantly higher in Korla pear (0.81 mg/kg FW) than in Nanguoli (0.41 mg/kg FW) or Jingbaili (0.39 mg/kg FW).

### 3.4 Changes of SSC and texture of pears during storage

#### 3.4.1 SSC

**Fig. 4:**
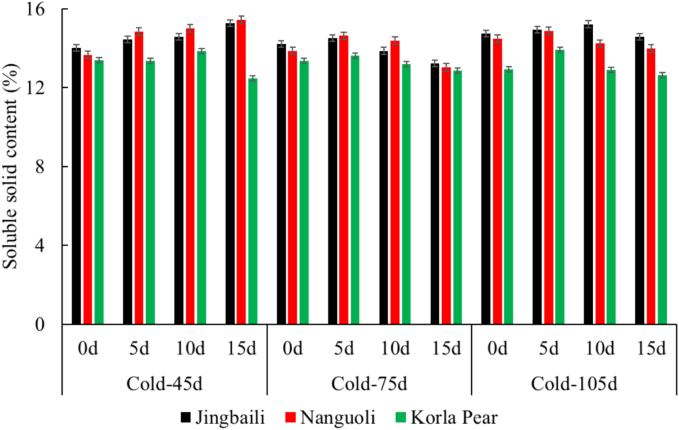
Dynamic changes of SSC in pear fruits with three representative cultivars during storage. Note: Firmness 1 and Firmness 2 are the peak forces recorded during the first and second compression cycles of TPA, respectively; cold means cold storage at 0 ± 1 °C; 0–15 d means shelf life at room temperature after cold storage.

SSC, which serves as the primary determinant of fruit sweetness, critically influences consumer preference and commercial value by directly reflecting sugar concentration (Harker et al., 2008; Wang et al., 2025). Throughout storage, SSC levels ranged from 13.67% to 15.43% for Nanguoli, 13.24% to 15.26% for Jingbaili, and 12.47% to 13.91% for Korla pears (Fig. 4). Comparable SSC levels were observed between Nanguoli and Jingbaili, both of which were consistently higher than those recorded in Korla pears. SSC in pear fruits (12.93%–14.75%) were stabilized during cold storage, which was primarily attributed to suppression of respiratory metabolism and enzymatic degradation of complex carbohydrates under low temperature. During the subsequent 15-day shelf life at ambient temperature, SSC exhibited a characteristic initial increase followed by a decline. The SSC of pear fruits during the shelf life generally reached its peak value on day 10 and 15.

#### 3.4.2 Texture

**Fig. 5:**
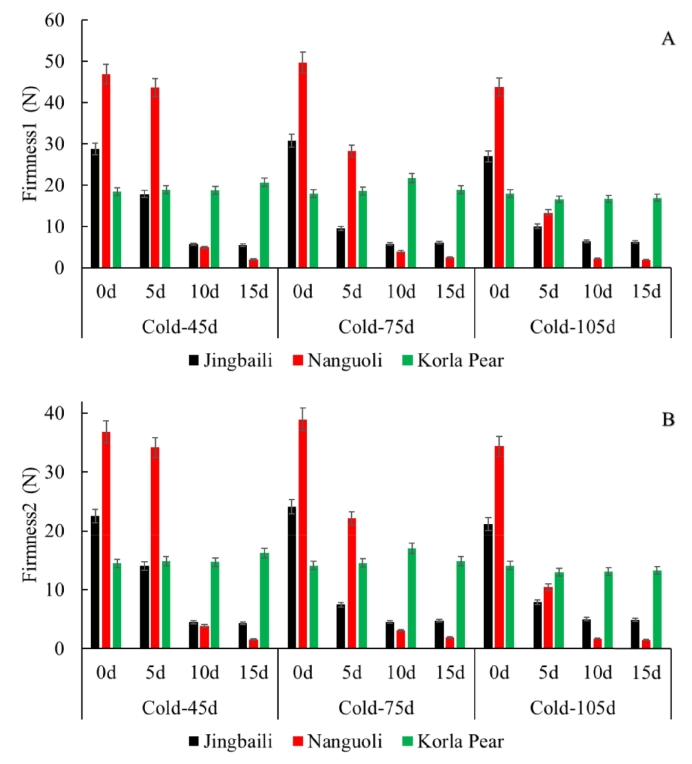
Dynamic changes of firmness in Nanguoli, Jingbaili, and Korla pear fruits during storage. Note: *indicates significance at *p* < 0.05; color depth indicates Pearson correlation coefficient values.

Fruit firmness is considered a critical quality parameter, directly influencing storability, transport tolerance, maturity assessment, sensory attributes, and processing suitability. In this study, Firmness 1 was conventionally employed to assess raw firmness and maturity. Firmness 2 reflected the residual structural strength of fruit tissue after the initial deformation and serves as an indicator of internal structural integrity and recovery capacity. During 105 days of cold storage, Nanguoli, Jingbaili and Korla pears maintained Firmness 1 values of approximately 50, 30, and 20 (Fig. 5A), respectively, while Firmness 2 values remained approximately 40, 20, and 15 (Fig. 5B). However, firmness of Nanguoli and Jingbaili decreased markedly to approximately 10%–20% of initial values by shelf day 10. This rapid softening was likely attributed to accelerated hydrolysis of pectin and hemicellulose in the cell wall matrix (Sun et al., 2023). In contrast, firmness of Korla pear maintained stable throughout 15 days of shelf life, demonstrating enhanced storage tolerance.

TPA was employed to further elucidate other textural dynamics during storage. As shown in Fig. S3, the structural integrity of all pear cultivars was effectively preserved by cold storage for 105 days, and values for fracture force, adhesiveness, cohesiveness, springiness, gumminess, and chewiness of pear fruits remained within narrow ranges (Nanguoli: 36.08–41.45 N, 0.14–0.22 N·mm, 0.11–0.12, 1.47–1.53 mm, 5.03–6.13 N, 7.53–9.47 mJ, respectively; Jingbaili: 25.35–27.60 N, 0.20–0.40 N·mm, 0.13–0.14, 1.45–1.55 mm, 3.46–4.17 N, 5.26–6.56 mJ, respectively; Korla pear: 16.22–17.32 N, 0.17–0.38 N·mm, 0.10–0.11, 1.05–1.16 mm, 1.77–1.93 N, 1.86–2.24 mJ, respectively). During the 15-day shelf life, rapid degradation across all TPA parameters was observed in Nanguoli and Jingbaili, suggesting advanced tissue melty and loss of cellular adhesion. In contrast, Korla pear maintained stable TPA parameters (except for adhesiveness), which reinforced its exceptional postharvest resilience.

### 3.5 Analysis of key VOCs in pear based on OPLS-DA and HCA

#### 3.5.1 Relationships between VOCs and quality attributes

**Fig. 6:**
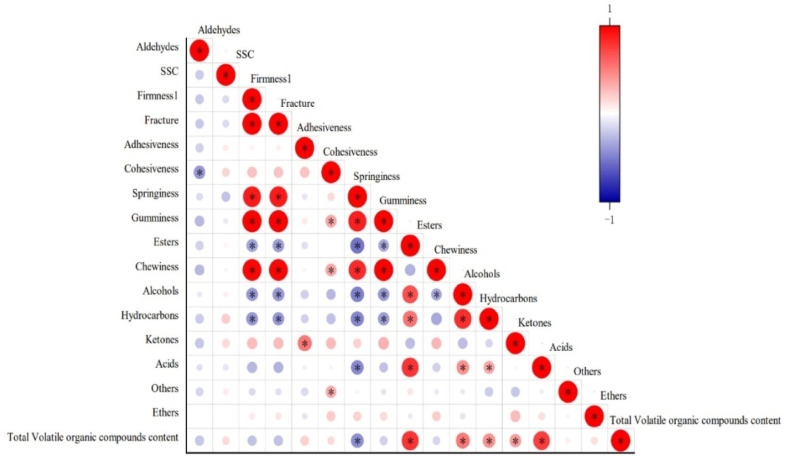
Correlation analysis between SSC, texture and total content of each volatile substance. Note: A, OPLS-DA score plot; B, OPLS-DA VIP score plot.

Analysis of Pearson correlations revealed significant interrelations among VOCs and their associations with key quality parameters (Fig. 6). Esters showed a strong positive correlation with alcohols, consistent with a potential biosynthetic relationship where alcohols act as precursors for ester formation via enzymatic esterification (Lara et al., 2003). Conversely, a significant negative correlation was observed between esters and aldehydes, aligning with the metabolic shift from aldehyde intermediates toward ester production during ripening (Espino-Diaz et al., 2016). Ketones displayed moderate positive correlations with both aldehydes and alcohols, suggesting shared biochemical pathways or co-regulation under the applied storage conditions. Collectively, these correlation patterns highlight the dynamic interplay within the VOCs profile, characterized by precursor-product dynamics and competitive metabolic fluxes.

Distinct associations were detected between specific classes of VOCs and key quality parameters (Fig. 6). Ester accumulation was strongly negatively correlated with firmness, fracture force, cohesiveness, gumminess, and chewiness. It implied that textural degradation facilitated ester production, likely through enhanced substrate accessibility for aroma biosynthesis enzymes following cell wall disassembly (Dheilly et al., 2016). Alcohols and aldehydes showed significant positive correlations with SSC, indicating their potential roles as markers of metabolic maturity (Zhang et al., 2022). Notably, ketones exhibited no significant association with textural parameters but displayed a weak positive correlation with SSC. The inverse relationship between textural integrity (firmness, fracture force, cohesiveness) and esters underscores the trade-off between tissue softening and aroma development during postharvest storage.

#### 3.5.2 OPLS-DA model discrimination and differential identification of VOCs

**Fig. 7:**
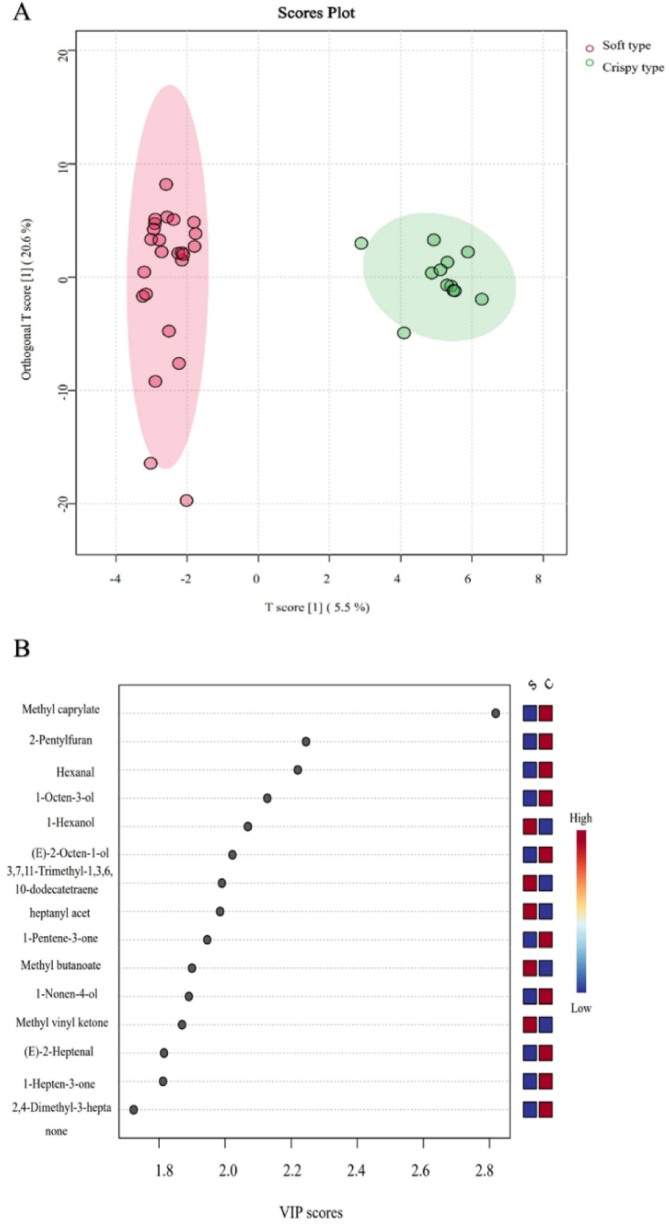
OPLS-DA analysis of 301 VOCs in pear fruits with different texture types.

Variable importance in projection (VIP) in OPLS-DA reflects the contribution of metabolite differences to sample group classification. Metabolites with VIP > 1 and *p* < 0.05 are considered significantly differential (Jiang et al., 2023). The OPLS-DA model effectively discriminated crisp-fleshed and soft-fleshed pear cultivars, exhibiting robust accuracy and stability (goodness-of-fit R2Y = 0.972, predictive ability Q2 = 0.641) (Fig. 7A). As can be seen in Fig. 7B, a total of 85 VOCs, predominantly aldehydes, ketones, and specific esters, with VIP > 1 were identified as key discriminators of flesh texture types, including methyl octanoate, 2-pentylfuran, hexanal, 1-octen-3-ol, 1-hexanol, and (E)-2-octen-1-ol. These VIP-ranked VOCs implicated coordinated shifts in lipid peroxidation, oxidative metabolism, and ester turnover during textural modification. Targeting these compounds in future studies may elucidate mechanistic drivers of texture deterioration and inform strategies for postharvest quality preservation.

#### 3.5.3 Correlation analysis and PCA

As indicated by prior work, significant correlations were observed between ester, alcohol VOCs and key textural attributes, including Firmness 1, fracture force, springiness, and gumminess. Therefore, ester and alcohol VOCs with VIP > 1 were selected for Pearson correlation analysis with these four textural traits (Fig. S4). Nine differential compounds were identified through this analysis, including 2-methylbutyl hexanoate, ethyl acrylate, 2-methylbutyl acetate, propyl hexanoate, pentyl acetate, 1-hexanol, (E)-2-octen-1-ol, 1-nonen-4-ol, and pentanol.

Subsequent PCA of the selected compounds and textural traits yielded six eigenvalues exceeding 1. Principal component 1 (PC1) accounted for 28.5% of the variance, while PC2 accounted for 25.7%. Together, these components explained 54.2% of the total variation. Distinct loading patterns were observed in the PCA biplot (Fig. S5A). Textural parameters exhibited strong positive loadings on PC1 and PC5, indicating co-variation. Esters were predominantly loaded on PC2 and PC5. Significant positive loadings for alcohols were distributed across PC2, PC3, and PC4.

Cluster analysis based on loading values segregated the variables into four distinct groups. All textural attributes were clustered together, confirming their interrelated nature. Three separate clusters were formed by the aroma compounds, with 2-methylbutyl hexanoate isolated as a distinct entity. This isolation suggested unique metabolic behavior for this compound. Clear varietal differentiation was revealed in the PCA score plot (Fig. S5B). Korla pear samples formed a tight cluster, indicating phenotypic consistency. Significant separation was observed between Korla and both Nanguoli (red) and Jingbaili (black) pears. The close proximity of Nanguoli and Jingbaili, which both belong to the *Pyrus ussuriensis* (Ussurian pear) taxonomic group, was noted. This spatial relationship reflected their shared genetic lineage and similar textural-volatile profiles. Collectively, these patterns suggested a cultivar-dependent mechanistic relationship between specific signatures of VOCs and textural properties in pear fruits.

## 4 Conclusion

In this study, dynamics of VOCs, SSC, and textural attributes in Nanguoli, Jingbaili, and Korla pears, during 105 days of cold storage (0 ± 1 °C) and a subsequent 15-day shelf life at ambient temperature was investigated. Comprehensive profiling of VOCs using SPME combined with GC–MS identified 301 compounds, predominantly esters, alcohols, ketones, and aldehydes. Pronounced increases in ester and alcohol VOCs diversity and contents, particularly in Nanguoli and Jingbaili, were observed during prolonged cold storage, with the most dramatic ester surge (25.96- to 113.55-fold) occurred after 75 days of cold storage. During shelf life, ester concentrations further escalated, especially following 75-day cold storage. SSC levels in pear fruits were stabilized during cold storage but exhibited an initial increase followed by a decline during shelf life. Obvious textural degradation, characterized by rapid declines in firmness and TPA parameters, occurred in the soft-fleshed cultivars (Nanguoli, Jingbaili) during shelf life. In contrast, Korla pears demonstrated exceptional textural stability throughout both storage phases, maintaining over 90% firmness retention and stable TPA parameters.

Multivariate statistical analyses (OPLS-DA, PCA, HCA) and Pearson correlation revealed critical interrelationships and discriminant markers. A strong negative correlation (*r* < −0.8) was consistently observed between the accumulation of ester VOCs and textural integrity parameters (firmness, fracture force, cohesiveness, gumminess, chewiness), underscoring a fundamental trade-off between tissue softening and aroma development. The OPLS-DA model effectively discriminated crisp-fleshed (Korla) from soft-fleshed pears, identifying 85 key discriminant VOCs (VIP > 1), including aldehydes (e.g., hexanal), ketones, and specific esters (e.g., methyl octanoate, 2-pentylfuran), implicating coordinated shifts in lipid peroxidation, oxidative metabolism, and ester turnover during textural modification. PCA further confirmed distinct cultivars clustering based on genetic lineage and textural-volatile profiles. These findings elucidate the cultivar-specific mechanistic links between biosynthesis pathways of VOCs and textural properties during postharvest storage. The identification of key discriminant VOCs and the quantitative relationships established between dynamics of VOCs and quality parameters provide actionable insights for optimizing tailored storage to balance aroma development with texture retention and for targeting these traits in pear breeding programs aimed at quality improvement.

## CRediT authorship contribution statement

**Guanwei Gao:** Writing – original draft, Investigation, Formal analysis. **Chen Yin:** Software, Methodology, Investigation, Conceptualization. **Luming Tian:** Writing – review & editing, Supervision, Project administration, Funding acquisition, Conceptualization. **Haifei Li:** Validation, Methodology. **Hongliang Huo:** Validation, Software. **Dan Qi:** Methodology, Investigation. **Ying Zhang:** Validation, Project administration. **Chao Liu:** Validation, Investigation.

## Declaration of competing interest

The authors declare that they have no known competing financial interests or personal relationships that could have appeared to influence the work reported in this paper.

## Data Availability

Data will be made available on request.
