# Supplementary material for: Cultivar-dependent interplay between volatile biosynthesis and texture deterioration in pear during cold storage and shelf-life
Source: Food Chem X. 2026 Apr 5;35:103837. doi: 10.1016/j.fochx.2026.103837 (PMC13091164; doi:10.1016/j.fochx.2026.103837)
Supplement: Supplementary file 1 — Supplementary material [file mmc1.docx]

**Supplementary Materials**

**Cultivar-Dependent Interplay between Volatile Biosynthesis and Texture Deterioration in Pear during Cold Storage and Shelf-Life**

Guanwei Gao^1, 3^, Chen Yin^1, 2^, Luming Tian^1, 2*^, Haifei Li^1, 3^, Hongliang Huo^1, 2^, Dan Qi^1, 2^, Ying Zhang^1, 2^, Chao Liu^1, 2^

1. Institute of Pomology, Chinese Academy of Agricultural Sciences, Xingcheng 125100, China
2. Key Laboratory of Germplasm Resources Utilization of Horticultural Crops, Ministry of Agriculture and Rural Affairs, Xingcheng 125100, China
3. Laboratory of Quality & Safety Risk Assessment for Fruit, Xingcheng 125100, China

*Correspondence: tianluming@caas.cn

**Table of content**

[**Fig. S1.** Composition of VOCs in pear fruits. 3](#_Toc223943349)

[**Fig. S2.** Comparison of composition of VOCs in pear fruits with various varieties. 4](#_Toc223943350)

[**Fig. S3.** Dynamic changes of textural properties of pear fruits during storage. 5](#_Toc223943351)

[**Fig. S4.** Pearson correlation coefficients between texture traits and VOCs. 6](#_Toc223943352)

[**Fig. S5.** PCA analysis based on texture traits and VOCs. 7](#_Toc223943353)

# **Fig. S1.** Composition of VOCs in pear fruits.


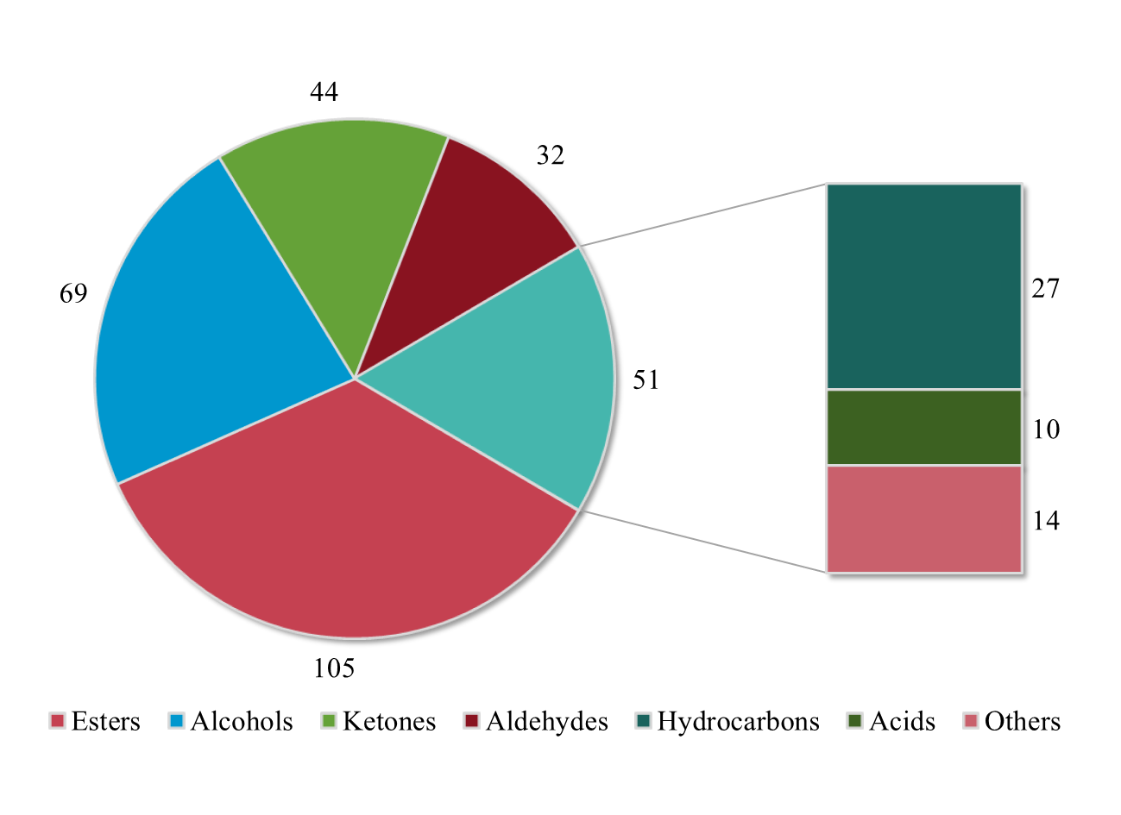


**Fig. S1.** Composition of VOCs in pear fruits with three representative cultivars.

# **Fig. S2.** Comparison of composition of VOCs in pear fruits with various varieties.


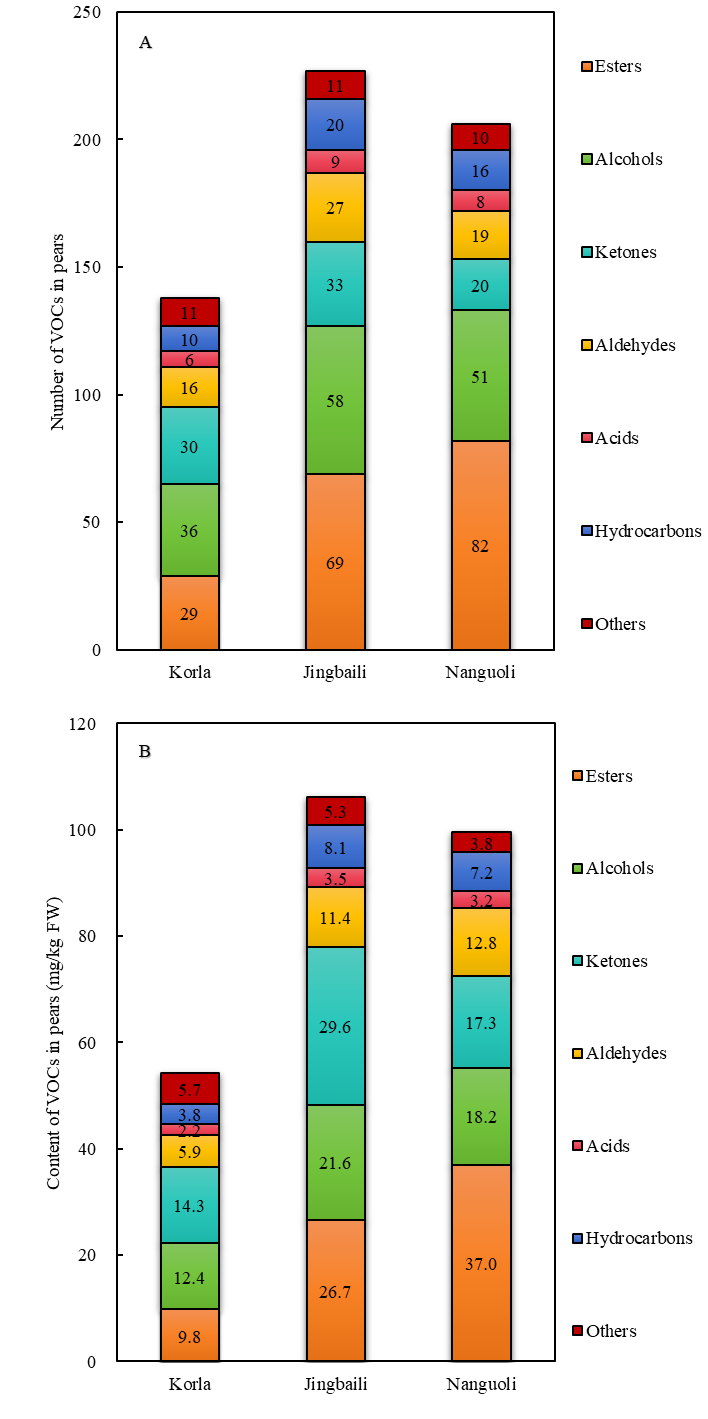


**Fig. S2.** Comparison of the types (A) and contents (B) of VOCs in the fruits of three pear varieties.

# **Fig. S3.** Dynamic changes of textural properties of pear fruits during storage.


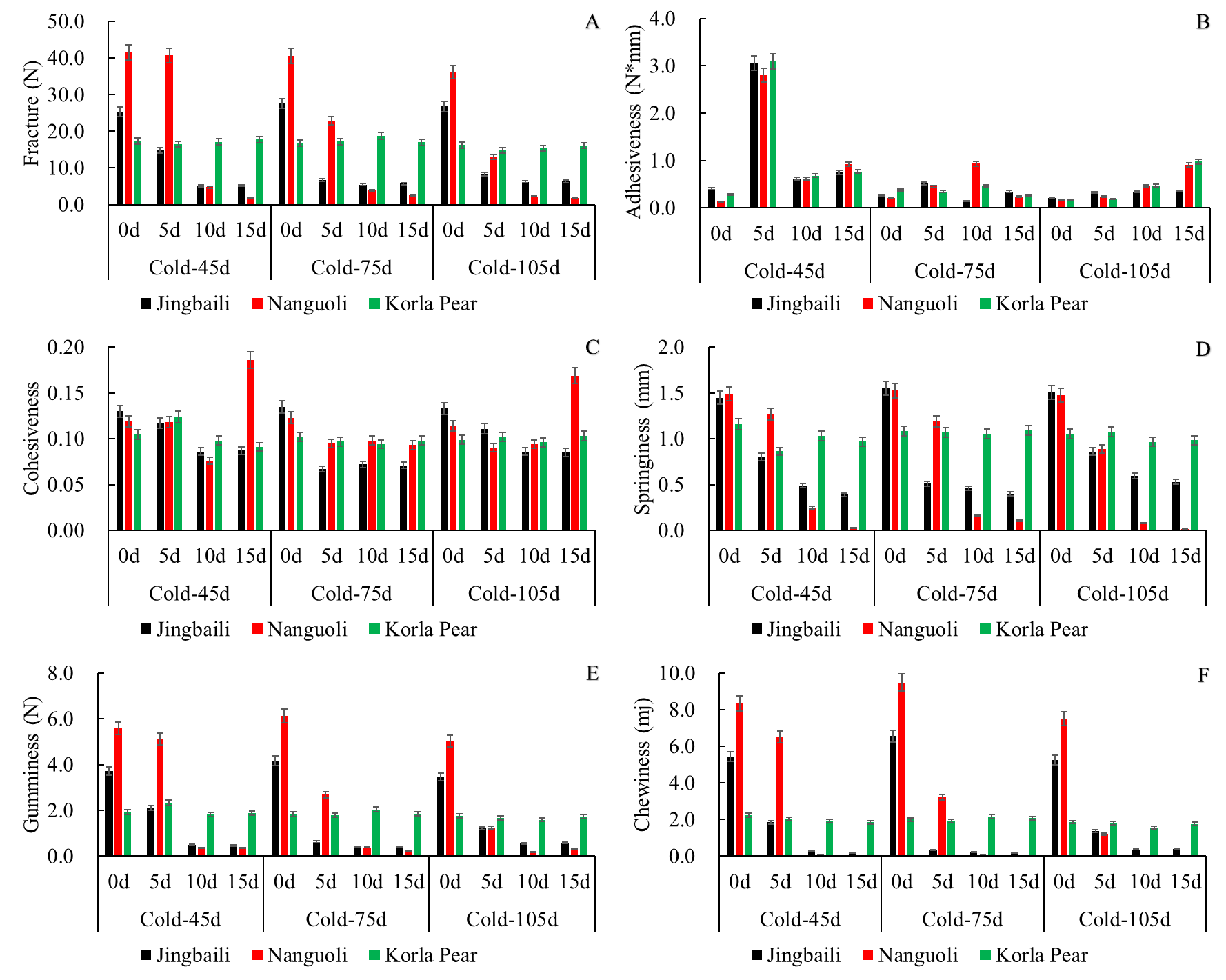


**Fig. S3.** Dynamic changes of fracture (A), adhesiveness (B), cohesiveness (C), springiness (D), gumminess (E), and chewiness (F) for pear fruits during storage.

# **Fig. S4.** Pearson correlation coefficients between texture traits and VOCs.


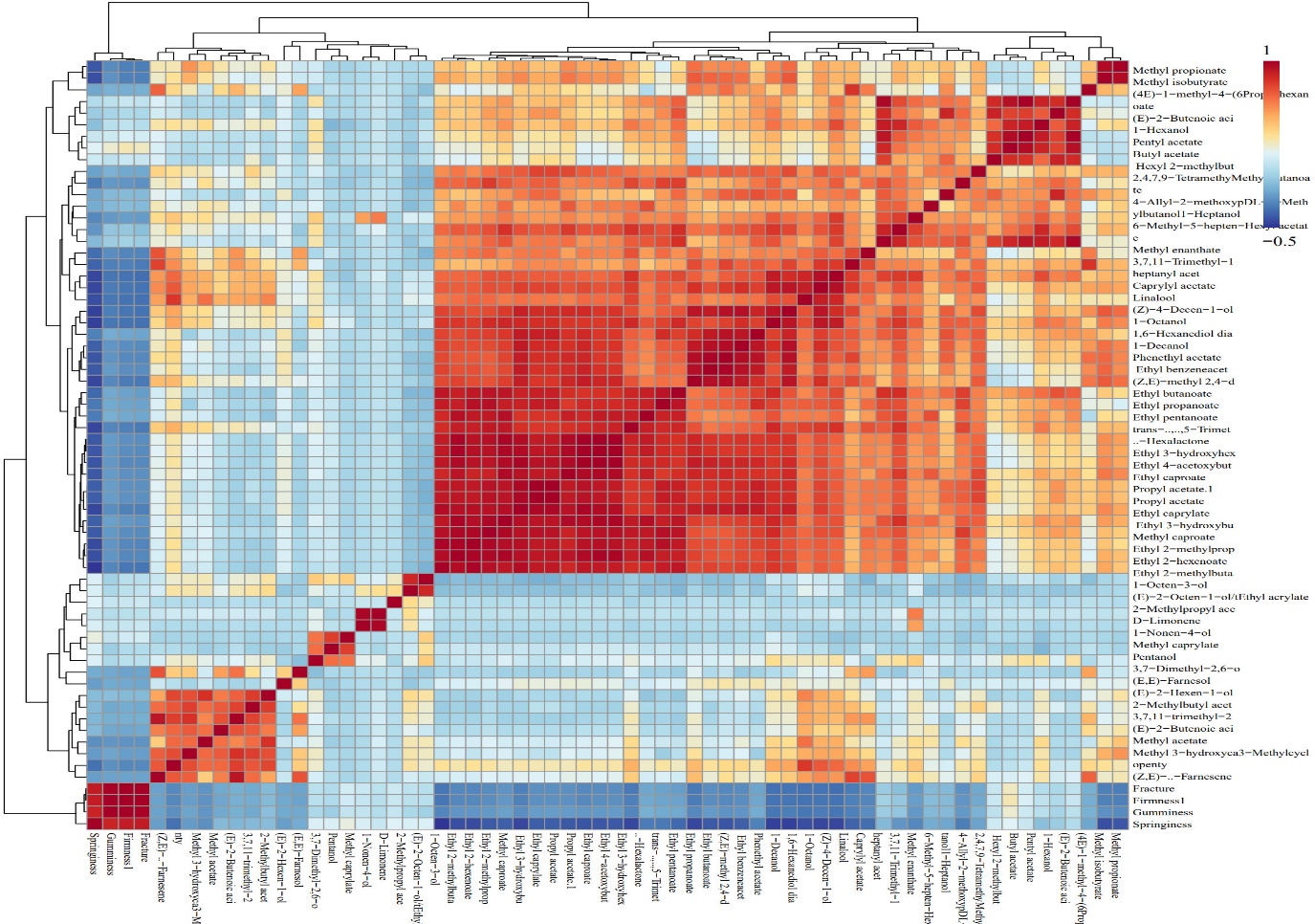


**Fig. S4**. Pearson correlation coefficients between selected texture traits and ester, alcohol compounds with OPLS-DA VIP values exceeding 1.

# **Fig. S5.** PCA analysis based on texture traits and VOCs.


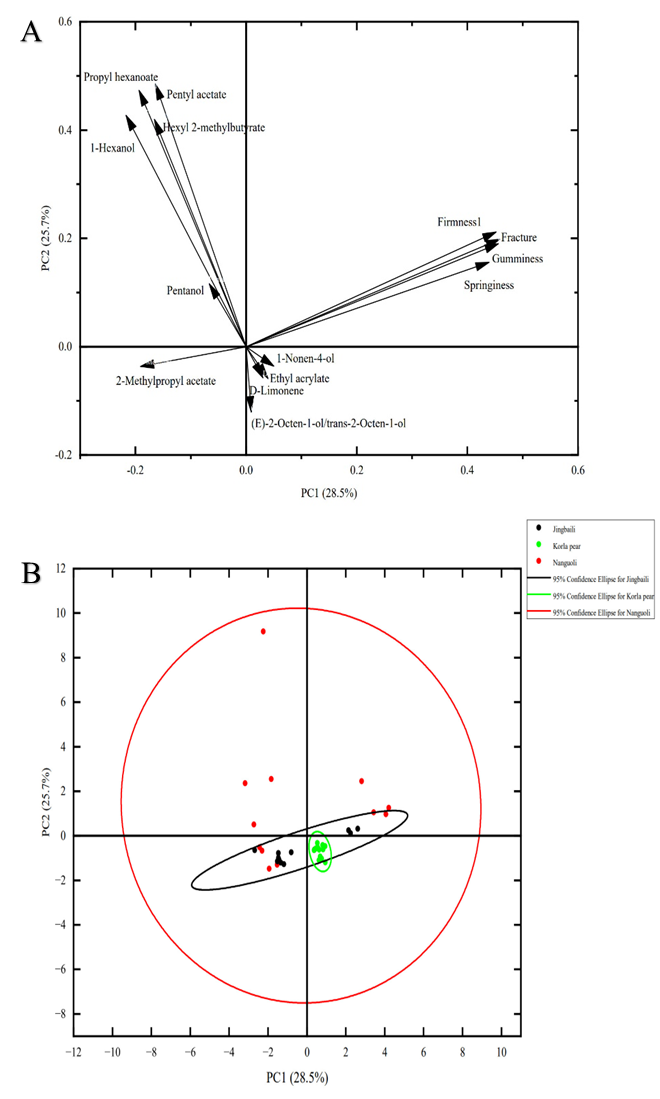


**Note:** A: PCA analysis plot; B: PCA comprehensive score plot, black represents Jingbaili, green represents Korla pear, and red represents Nanguoli pear.

**Fig. S5**. PCA analysis based on the selected texture traits and VOCs.
